# Case of Tic Douloureux, Successfully Treated by Carbonate of Iron

**Published:** 1823-09

**Authors:** John Evans Beale

**Affiliations:** Member of the Royal College of Surgeons.


ART. VI.—*Case of Tic Douloureux, successfully treated by Carbonate of Iron.* By JOHN EVANS BEALE, Esq. Member of the Royal College of Surgeons.

So many successful cases of the treatment of tic douloureux by means of the carbonate of iron, as recommended by Mr. Hutchinson, of Southwell, have been noticed in your excellent Journal, as to render a single instance of its efficacy almost superfluous; yet the concurring testimony of every medical man must be of value, which goes to prove that this hitherto intractable disease is within the controlling powers of medicine: for it is upon such evidence, rather than upon a number of favourable results communicated by one individual, such a degree of confidence is insured among our professional brethren as may lead to the general adoption of any remedy. In my own mind, I have not the smallest doubt but that, by such means, a body of facts will be established in favour of the use of iron in cases of tic douloureux as to lead to its general employment. That it is a specific, Mr. Hutchinson is, I am sure, too scientific a practitioner to assert: daily experience proves to us that no medicine, in any disease whatever, merits such a title; whilst, to call it such, would shake our confidence in the remedy to which it might relate; but that this is one of the most fortunate discoveries, in curing a disease formidable alike from its obstinacy and its violence, the following case will, I hope, help to prove.

John Mannock, aged fifty-five, of spare habit, but healthy constitution, applied to me on the 13th of May, 1822, complaining of severe paroxysms of pain extending over the whole of one side of the face, particularly affecting the lips; and hence their approximation, in eating or speaking, was attended with most severe suffering: the sensations he compared to an electric shock, lasting as it were for a minute, and repeated as often as twenty or thirty times in an hour. The whole of the branches of the fifth pair of nerves, as distributed over the affected side of the face, were evidently the seat of pain. He was unable to assign any cause for the disease. As the tongue indicated disturbance in the digestive functions, a saline purging mixture was directed to be taken at intervals in the day; calomel combined with antimony and opium, at bed-time; and leeches were applied to the face. These medicines were repeated on the 14th, with the addition of hyoscyamus. On the 15th, blisters were applied behind the ears, and fomentations employed to the face. The leeches were also repeated on the 16th. On the following day, far from being relieved, he declared the torment he suffered to have become absolutely insupportable; and his tongue was more furred than when I saw him first. On the 17th he commenced taking the carbonate of iron, in doses of two scruples three times a-day: after the first twenty-four hours, he stated the pain as diminished, both in force and frequency; and, having continued the powders to the 29th of the same month, he was at that time entirely free from pain. He is a man of temperate habits, and accustomed to regular exercise. The circumstance of the gradual improvement of the tongue in this case under the administration of iron, would induce me to believe the disordered state of the stomach is not to be regarded uniformly as the cause, but sometimes as the consequence, of this affection.

*Plaistow, Essex; July* 1823.

